# National assessment of pharmaceutical workforce and education using the International Pharmaceutical Federation’s global development goals: a case study of Qatar

**DOI:** 10.1186/s40545-021-00305-y

**Published:** 2021-02-22

**Authors:** Banan Abdulrzaq Mukhalalati, Meram Mohamed Mahmoud Elsayed Ibrahim, Majdoleen Omar Al Alawneh, Ahmed Awaisu, Ian Bates, Lina Bader

**Affiliations:** 1 Clinical Pharmacy and Practice Section, College of Pharmacy, QU Health, Qatar University, P.O. Box 2713, Doha, Qatar; 2 College of Pharmacy, Qatar University, Doha, Qatar; 3 College of Pharmacy, QU Health, Qatar University, P.O. Box 2713, Doha, Qatar; 4 UCL School of Pharmacy, Director of Education Development in FIP, UCL, London, UK; 5 FIP Lead for Workforce Transformation and Development, International Pharmaceutical Federation, The Hague, Netherlands

**Keywords:** International Pharmaceutical Federation, Pharmaceutical Workforce Development Goals, Workforce development, Delphi, Pharmaceutical workforce and education, Pharmacy education and practice

## Abstract

**Background:**

The sustainable development goals were launched by the United Nations in 2015. Its fifth goal was describing the achievement of universal health coverage by 2030. This goal reaffirms the importance of investing in the development and training of the global health workforce. In alliance with this, the International Pharmaceutical Federation (FIP) has published reports about pharmacy workforce planning in several countries. However, data about Qatar were not included in these reports. In 2017, FIP developed a transformational roadmap of pharmaceutical workforce and education. One systematic framework component of the roadmap is the Pharmaceutical Workforce Development Goals (DG[w]s) that were released in late 2016 and subsequently incorporated into the more comprehensive Global Development Goals^1^ in 2020, encompassing not only workforce development, but additionally practice and pharmaceutical science development. This study aimed to evaluate the current situation of pharmacy workforce and education in Qatar in relation to the original 13 Pharmaceutical Workforce Development Goals (DG[w]s). The objective was to identify the gaps in pharmacy workforce and education and to recommend evidence-led strategies to be included in both the Ministry of Public Health and the Qatar University College of Pharmacy workforce development plans.

**Methods:**

Three rounds of conventional Delphi technique were conducted with expert panels of key decision-makers in pharmacy practice from the College of Pharmacy at Qatar University and the Ministry of Public Health, utilizing the FIP’s self-assessment survey. Qualitative content analysis was used to analyze and prioritize the identified gaps from the collected data. DG[w] was considered “met” if all the provided indicators were achieved, “partially met” if at least one of the indicators were achieved, and “not met” if none of the indicators were achieved

**Results:**

The lack of competency framework (DG[w]5), workforce data (DG[w]12), and workforce policy formation (DG[w]13) are three major gaps in the provision of pharmaceutical workforce and pharmacy education in Qatar, influencing other DG[w]s. These gaps need to be addressed by the formation of Qatar Pharmaceutical Association through which academic, practice, and policymaking sectors can work together in developing health workforce intelligence system.

**Conclusion:**

The results indicated that DG[w]s are interrelated and a gap in one goal can negatively influence others. Results and recommendations of this research will facilitate the implementation of strategic plans across leading pharmacy sectors to meet health needs in Qatar and achieve the third pillar of the Qatar National Vision 2030.

## Introduction

The United Nations launched the sustainable development goals in 2015, which are global development targets, to be achieved by 2030—building on the millennium development goals that preceded them; the fifth sustainable development goal describes achieving universal health coverage including access to quality essential healthcare services for all citizens by 2030 [1].^1^ This goal reaffirms the importance of investing in the development and training of the global health workforce especially as the availability of a capable, competent and accessible healthcare workforce is now a global challenge [1–3], which is a potential barrier to the improvement and development of national and global health services [4]. Hence, health workforce planning is a critical requirement in global healthcare agenda in an effort to achieve universal health coverage [5, 6].

Pharmacists have a key role in the delivery of primary and secondary healthcare [7], and factors such as increased complexity of medication therapies, increased complexity of co-morbidities and number of prescriptions have contributed to the demand of pharmacy workforce across all global regions [6]. Consequently, new entrants into the pharmacy workforce need to possess the skills and competencies needed to deliver efficient pharmaceutical and health services as part of the healthcare team [8]. Several substantial changes are required to be implemented by pharmacy schools, associations and employers in order to ensure a balance of demand and supply in the pharmacy workforce [9]. Analyses of the global pharmacy workforce have consistently showed that countries and territories with lower economic indicators tend to have fewer pharmacists and pharmacy technicians [10]. However, while the quantitative capacity of the pharmacy workforce is important, it is not the only factor: the ability of this workforce to meet the healthcare needs of populations is of equal importance [11].

The capacity of the pharmacy workforce varies both within and across the six World Health Organization (WHO) regions. For example, the estimated pharmacist density in northern countries in the region of the Americas, such as Canada and United States of America, are between 9.8 and 10.5 pharmacists per 10,000 population [12]. Countries in the Africa region tend to have fewer pharmacists per population compared to other regions; for example, pharmacist density is as low as 0.02 pharmacist per 10,000 population in Somalia [10]. In the Eastern Mediterranean Region (EMR), pharmacy practice continues to evolve coupled with highly varied capacity densities between countries within the region [13]. Recent estimated pharmacist densities in the EMR range from 1.6 (Bahrain) to 16.2 (Jordan) pharmacists per 10,000 population [12]. Furthermore, some countries in the EMR such as Jordan are considered as “exporters” of pharmacists to neighboring countries since the number of graduates are considered to exceed the need for that particular country but address the shortages of others [12]. For example, countries like Iraq and Bahrain are examples of countries with low pharmacist densities (3.3 and 1.6 per 10,000 population, respectively) where shortages of local graduates exists, and thus expatriate pharmacists are hired [12]. Other countries such as Qatar, United Arab Emirates and Saudi Arabia have moderate pharmacist density which are 8.9, 8.8 and 8.6 per 10,000 population, respectively.

The International Pharmaceutical Federation (FIP) is the global body representing the pharmacy profession including pharmaceutical sciences, practice and education. Through more than 140 national pharmacy organizations across the globe, FIP represents over four million pharmacists, pharmaceutical scientists and pharmaceutical educators worldwide [14]. In 2017, FIP published an analysis of trends in the global pharmacy workforce at three time points (years 2006, 2009 and 2012) providing baseline data on the current global trend regarding the number of pharmacists, capacity building, and mean percentage change in the pharmacist capacity [7]. The report indicated a shortage of pharmacy workforce in the low and middle-income countries [7] which includes many of the countries in the EMR. However, most of the available data regarding pharmacy workforce are from the regions of the Americas and Europe, despite the coverage of all six regions in the report [15]. It is worth noting that workforce planning literature from the United Kingdom, United States of America, and Canada have discussed the expected challenges in the pharmacy profession and have proposed potential solutions and actions [16, 17]. In these countries, actions have been taken and reports regarding these challenges have been sent to regulatory bodies in order to address the identified challenges [16, 17]. Profound professional actions were taken by the United Kingdom. These actions include inclusive workforce assessment and subsequent pharmacy stakeholders’ participation in workforce planning, which was followed by provision of potential solutions to the profession and public sectors. One consequence, coupled with a general market expansion of higher education, has been a large increase in the number of pharmacy schools, faculties and colleges over the previous 20 years [16, 17].

In EMR, studies conducted to address workforce intelligence and workforce development strategies are limited and the available published literature emphasizes workforce statistics without analyses of workforce planning and intelligence [18, 19]. For example, a published review in 2011, which included 13 EMR countries, reported that the number of pharmacists and pharmacy educational programs were increasing in the region, but did not provide suggestions about the effective utilization and development of the workforce [13]. In one EMR country—Qatar, the available literature suggested several challenges facing the pharmacy profession, including the absence of a national pharmacy association [11]. However, the literature emphasized that the support from the Qatar government through the Ministry of Public Health (MoPH) represents an opportunity for development in alignment with the Qatar National Vision 2030 [11, 20].

In 2017, FIP led the establishment of a global developmental roadmap that aims to transform pharmaceutical education and workforce by providing appropriate strategic tools to support and develop quality-driven pharmacy education and workforce planning [21]. FIP transformative workforce roadmap, which was developed by collaborative work and consensus from global stakeholders of pharmacy, is an evidence-generated roadmap with a global vision for transforming pharmacy [22]. One of the major components of the global roadmap are 13 global Development Goals specifically related to workforce (DG[w]s) grouped into three main clusters: academic, professional development, and systems, as illustrated in Appendix 1. The academic cluster includes three goals, focusing on schools, universities and education providers, and aims to assess academic capacity, foundation training and early career development and quality assurance. The professional development cluster includes five goals, focusing on the pharmaceutical workforce. The five goals aim to assess advanced and specialist expert development, competency development, leadership development, service provision and workforce education and training, and working with others in the healthcare team. Finally, the systems cluster includes five goals, focusing on policy development, governmental strategy planning and monitoring systems. The systems cluster goals aims to assess continuing professional development strategies, pharmaceutical workforce gender and diversity balances, workforce impact and effect on health improvement, workforce intelligence and workforce policy formation [21]. DG[w]s are considered a mapping framework and, hence, do not prioritize the proposed goals; instead, prioritization is left for local stakeholders, based on their individual country’s needs [21].

This study represents the first national-level research-oriented response in the EMR to FIP’s call for further concerted effort towards national cohesive workforce development and education, following their publication, at the EMR regional level coupled with the local imperatives for alignment with national health and policy visions [23]. The aims of this study were to: (1) evaluate and assess the current situation of pharmacy workforce, education, profession and system from different institutional and stakeholder perspectives in the State of Qatar in relation to the 13 DG[w]s; (2) identify the gaps in pharmacy workforce, education and practice development and; (3) recommend possible solutions as potential strategies to be included in the Ministry of Public Health (MoPH)’s and College of Pharmacy’s (CPH) at Qatar University (QU) future plans.

## Methodology

### Study design

A conventional Delphi method was applied in this research study. This method was selected because it is a structured communication method for achieving consensus with target participants by using iterative rounds of questionnaires to collect data. The use of a conventional Delphi method in this research facilitated knowledge sharing and views broadening between the participants and stimulated the generation of new ideas about workforce development in Qatar [24–27]. This research method was adjusted in accordance with the current study’s proposed objectives about assessing the status of DG[w]s in Qatar, while maintaining the main features of the Delphi method [26, 28, 29].

### Population and sampling

Professional leaders and policymakers in the MoPH (*n* = 6) were identified as key decision-makers in pharmacy practice relating to workforce development and licensing in Qatar and were invited to participate in the study. These individuals have the ability to influence practice policies and workforce development. Additionally, policymakers and leading educators in the CPH (*n* = 5) at QU—the country’s only pharmacy college—were identified as key decision-makers in pharmacy education and were invited to participate in the study. These individuals have the ability to influence pharmacy education’s policies and development in Qatar.

### Study setting

This study was conducted in CPH at QU. The data were collected over 3 months between September and December 2018. The participants who were involved in the study received an information leaflet which included adequate information about the nature, purpose, and anticipated risk of the study. Written informed consent forms were obtained from the participants prior to their recruitment in the study.

### Delphi tool and study instrument

FIP’s validated self-assessment survey tool was used for data collection in this study in order to assess the alignment of pharmacy practice and pharmacy education in Qatar with the DG[w]s. The tool was used in a 21-country global survey study conducted by FIP in 2017; the tool was initially drafted and peer-reviewed internally within FIP. The template was then sent to two contacts from member organizations to pilot the questions. Comments on clarity of the questions and response options available were received and changes were made accordingly before being released worldwide [23].

### Data collection

Three rounds of Delphi were conducted; each round was more focused aiming to reach confirmation and consensus among participants. First Delphi round was done by sending the FIP’s validated self-assessment survey tool via e-mails to obtain individual responses (*n* = 6 from MoPH, *n* = 5 from CPH). Participants were asked to answer relevant question about DG[w]s related to their area of expertise (Participants from CPH answered questions related to academy cluster goals and participants from MoPH answered questions related to professional development and system cluster goals). Participants then sent the completed questionnaires back to the researcher by email, in order to retrieve and organize the collected data. In the second Delphi round, the researcher invited participants from MoPH (*n* = 6) to a meeting and participants from CPH (*n* = 5) to another meeting in order to conduct around the table detailed discussion about participants’ collected answers from the first round and their interpretation in order to elaborate further about their answers and reach consensus (consensus was ≥ 90% for all questions). The third Delphi round involved sending the consensed upon answers by email to each participant, who were asked to confirm their agreement on the reached consensus. Summary for the three rounds of the Delphi approach’s participants is shown in Table 1. All data collection sessions were conducted in English language and were recorded using a digital audio recorder to allow the researchers to focus on discussions and consensus achievement. Recorded sessions were manually transcribed by the researchers using the verbatim transcription and were double-checked for accuracy in transcription.

**Table 1:** Summary for the three rounds of the Delphi approach with participants from the Ministry of Public Health and college of pharmacy

	Number of participants who responded (Round 1)	Number of participants who attended the meeting (Round 2)	Number of participants who established and confirmed the final consensus (Round 3)
MoPH	6	4	6
CPH	5	4	5

### Data analysis

A qualitative content analysis technique was used to analyze the collected data in this research. This method was chosen for analysis because it has the ability to focus on the context of the current situation of pharmacy practice and education in Qatar [30, 31]. Moreover, it highlights the similarities within, and the differences between, the different parts of the FIP validated survey and draws conclusions from the data [32, 33]. From the data collected, a goal was considered “met” if all the provided indicators were considered achieved by the participants’ consensus, “partially met” if at least one of the indicators were considered achieved by the consensus, and “not met” if none of the indicators were considered achieved by the consensus.

## Results

### Academy cluster

**Fig. 1:**
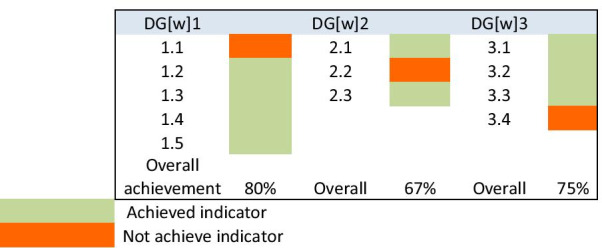
Percentages of indicators achievement in the academy cluster goals

All participants from CPH responded to the academy questions. The three goals in the academy cluster were “partially met”, as shown in Table 2, with each goal achieving more than half of its indicators (Fig. 1). It was reported by the participants from CPH that the present increase in academic capacity (DG[w] 1) was not based on national needs, meaning that one of its five indicators not achieved. One of the CPH’s participant responses, about this unachieved indicator, was as follows:

“I am not sure if there are serious discussions and strategic planning about workforce development agenda, plus any documents i.e. evidence that were shared at the college level regarding the needs of the country. Moreover, no proper manpower study for the pharmaceutical sector that was carried out and published/shared/circulated in CPH.” CPH3

**Table 2:** Summary of the responses of the indicators in each goal among the academy cluster

DG[w]1. Academic Capacity: Engagement with pharmaceutical higher education development policies and ready access to leaders in pharmaceutical science and clinical practice in order to support supply-side workforce development agendas		
Indicator	Participants’ consensus about the indicator achievement status	Assessment of the indicators
Increasing capacity to provide a competent pharmaceutical workforce according to national health resource needs	The college is planning to increase its capacity to provide competent pharmaceutical workforce in the clinical practice and pharmaceutical industry scopes. However, this increase in capacity is not based on national health resources needs	Not achieved
Develop new and innovative ways to attract young pharmacists into all areas of pharmaceutical practice and science	The college offers several degrees and Specialties; bachelor, PharmD, masters and PhD	Achieved
Capacity building should include the ability to meet minimum national standards of facilities, educators and student support	Capacity building meets the national standards of facilities, educators and student support	Achieved
Enhance interprofessional education and collaboration with key stakeholders, including governments, national and international pharmacy/pharmaceutical organizations and patient advocacy groups to achieve sustainable solutions for capacity development	The learning process encourages interprofessional education and collaboration with national stakeholders and international organizations is facilitated	Achieved
The clinical academic educator workforce needs more attention to training, career development and capacity building, which must, importantly, include research capacity enhancement	Research capacity enhancement is given the required attention by educator workforce in the college	Achieved

Overall goal status		Partially met
DG[w] 2. Foundation training and early career development: Foundation training infrastructures in place for the early post-registration (post-licensing) years of the pharmaceutical workforce as a basis for consolidating initial education and training and progressing the novice workforce towards advanced practice		
Indicator	Participants’ consensus about the indicator achievement status	Assessment of the indicators
Create clear and purposeful education and training pathways/programs to support post-registration (post-graduation) foundation training (clinical practice and pharmaceutical science areas)	Post-registration foundation training is supported by purposeful education and training programs	Achieved
Develop early career maps and frameworks to support a seamless transition into early career practice and towards advanced practice	The SPEP* rotations for students provide seamless transition towards practice. However, there are no frameworks and maps currently in the college to support such transition	Not achieved
Develop structured approaches to early career mentoring systems to support novice practitioners to engage with peers and preceptors (in clinical practice and pharmaceutical science areas across the pharmaceutical workforce)	The college support novice practitioners to engage with peers and preceptors through SPEP* rotations	Achieved

Overall goal status		Partially met
DG[w] 3. Quality assurance: Transparent, contemporary and innovative processes for the quality assurance of needs-based education and training systems		
Indicator	Participants’ consensus about the indicator achievement status	Assessment of the indicators
Ensure the quality of the workforce by quality assuring the continuous development and the delivery of adequate and appropriate education and training, quality assurance needs to address academic and institutional infrastructure in order to deliver the required needs and competency-based education and training	The quality assurance tasks in the college include; PLOA** and APR***	Achieved
Establish standards based global guidance for quality assurance of pharmacy and pharmaceutical science education in the context of local needs and practice	QU CPH has several layers for quality assurance, including CCAPP accreditation, committees and academic program reviews	Achieved
Implement fair, effective and transparent policies and procedures for quality assurance of pharmacy and pharmaceutical science education and training	CPH’s setting has representations from students and faculty to ensure transparency in quality assurance-related policies and procedures	Achieved
Define critical stakeholder input on development of adequate education and training and fair and effective policies, including necessary student input	Critical stakeholders (MoPH) do not have input on policy development, including student input, at the current time	Not achieved
Overall goal status		Partially met

*Structured Practice Experiences Program

**Program Learning Outcome Assessment ***Academic Program Review

According to CPH’s participants, DG[w]2, which focuses on foundation training and early career development, was partially met with one of its three indicators not achieved. This was due to the lack of early career maps and frameworks to support a seamless transition for the students into early career practice and towards advanced practice in the CPH. However, the first indicator of this goal, which focuses on the students’ foundational education and training was achieved. A CPH participants commented about this indicator, as follows:

“Yes, we are currently in alignment. Structured clinical practice training is embedded into our curriculum in both the BSc and PharmD programs which prepares students for practice”. CPH1

Study participants suggested that DG[w] 3, which focuses on the quality assurance processes, was partially met, with one of the four indicators not achieved. One possible reason for this indicator being not achieved is the absence of stakeholder input for training and education development. However, three indicators were achieved, for example, participants indicated that they have internal and external quality assurance at QU. For example, the following was mentioned by one of the CPH’s participants about the first achieved indicator:

“In 2016, we conducted an Academic Program Review, as requested by QU, where we invited two panel of experts from North America to review our curriculum”. CPH3

Another participant mentioned the following about the second achieved indicator:

“The CPH is in alignment with the quality assurance process. We are accredited by the Canadian Council for Accreditation of Pharmacy Programs for both BSc and PharmD. This accreditation ensures the quality of the education that is provided for our students”. CPH2

### Professional development cluster

**Fig. 2:**
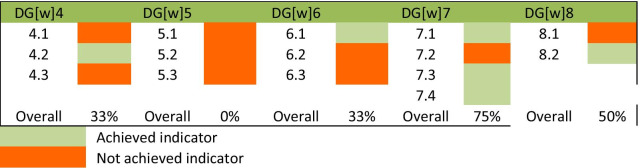
Percentages of indicators achievement in the professional development cluster goals

All participants from the MoPH responded to the professional development items. The professional development cluster includes five goals, for which one goal was “not met” and four goals were “partially met”. The responses on the professional development cluster are shown in Table 3 and the percentages of the achieved indicators for each goal are shown in Fig. 2. DG[w] 4, focussing on advanced and specialist expert development, was found by the study participants to be partially met with two of its three indicators not achieved. The evidence gaps were highlighting the need for a common and shared understanding of what is meant by “specialization training” and “advanced practice” in the context of scope of practice and the responsible use of medicines. About this indicator, a policymaker from the MoPH said:

“Qatar Council for Healthcare Practitioner as a regulatory authority does not conduct this kind of specialization training. However, number of accredited activities are conducted by healthcare organization and academic institution for pharmacists”. MoPH4

**Table 3:** Summary of the responses of the indicators in each goal among the professional development cluster

DG[w] 4. Advanced and specialist expert development: Education and training infrastructures in place for the recognized advancement of the pharmaceutical workforce as a basis for enhancing patient care and health system deliverables		
Indicator	Participants’ consensus about the indicator achievement status	Assessment of the indicators
Need for a common and shared understanding of what is meant by “specialization” and “advanced practice” in the context of scope of practice and the responsible use of medicines	CPD* programs aim to advance the level of the pharmaceutical workforce. However, there is no common and shared understanding of specialization and advanced practice	Not achieved
Ensure competency and capability of an advanced and expert pharmacist in all sectors for greater optimization of complex pharmaceutical patient care. This may now include prescribing roles within a recognized scope of practice	The State of Qatar requires all licensed healthcare practitioners including Pharmacists to participate in CPD* activities according to the policies and regulations established by the QCHP** Accreditation Department	Achieved
Systematic use of professional recognition programs/systems as markers for advancement and specialization across the workforce, including advanced pharmaceutical scientists	No professional recognition systems are used as markers for advancement across the workforce	Not achieved

Overall goal status		Partially met
DG[w] 5. Competency development: Clear and accessible developmental frameworks describing competencies and scope of practice for all stages of professional careers. This should include leadership development frameworks for the pharmaceutical workforce		
Indicator	Participants’ consensus about the indicator achievement status	Assessment of indicators
Use of evidence-based developmental frameworks to support the translation of pharmaceutical science within scope of practice, across all settings and according to local/national needs	Such evidence-based development frameworks are not existed	Not achieved
Support professional career development by using tools, such as competency frameworks, describing competencies and behaviors across all settings	There are competencies in the clinical and ethical domains for entry to pharmacy practice. However, it is not a framework for all professional careers’ stages	Not achieved
Evidence of clear policy that links leadership development (from early years) with competence attainment for the advancement of practice activities	No such evidences exist at the present time	Not achieved

Overall goal status		Not met
DG[w] 6. Leadership development: Strategies and programs in place that develop professional leadership skills (including clinical and executive leadership) for all stages of career development, including pharmaceutical sciences and initial education and training		
Indicator	Participants’ consensus about the indicator achievement status	Assessment of indicators
Creation of programs/strategies for the development of leadership skills (including tools and mentoring systems), to support pharmacists and pharmaceutical scientists through their careers	Programs for leadership development are offered and encouraged to attend for pharmacists and pharmaceutical scientists to support them in their careers	Achieved
Advocacy for leadership development in healthcare teams, linked to collaborative working activities	Programs for leadership development are delivered with encouragement to attend. However, they are not mandated	Not achieved
Ideally, this should be linked with competency and foundation and early year career development activities	Programs are not linked with competencies	Not achieved

Overall goal status		Partially met
(DG[w] 7. Service provision and workforce education and training: A patient-centered and integrated health services foundation for workforce development, relevant to social determinants of health and needs-based approaches to workforce development		
Indicator	Participants’ consensus about the indicator achievement status	Assessment of indicators
Systematic development of education and training activities based on local healthcare systems, their capacity and funding	The National CME***/CPD* Program and accreditation system promote patient-centered and local needs-based CPD* of all HCPs, including pharmacists	Achieved
Evidence of systematic development policies and strategies for the strengthening and transforming pharmaceutical workforce education and the systematic training of trainers/educators	No evidence of systematic development policies for transforming pharmaceutical workforce education	Not achieved
Education providers must ensure, by the provision of evidence-based approaches, that lecturers/teachers/trainers are themselves appropriately trained for capability and competency	The QCHP** has a national CPD* accreditation system that ensures the quality of CPD* providers against sets of standards	Achieved
Enable the pharmaceutical workforce and key stakeholders to promote health equity through actions related to social determinants of health	The CME***/CPD* accreditation system is intended to promote consistent quality and excellence in CME and professional development	Achieved

Overall goal status		Partially met
DG[w] 8. Working with others in the healthcare team: Clearly identifiable elements of collaborative working and interprofessional education and training which should be a feature of all workforce development programs and policies		
Indicator	Participants’ consensus about the indicator achievement status	Assessment of indicators
Evidence of policy formation to demonstrate How healthcare professionals can develop and engage in partnerships to achieve better health outcomes	There is no evidence of policy formation to demonstrate how healthcare professionals can develop and engage in partnerships for better health outcomes as these activities are not mandated	Not achieved
Develop education and training strategies/ programs to ensure collaboration within the pharmaceutical workforce and training on medicines for other healthcare professionals	Most strategies and programs are based on interprofessional education	Achieved
Overall goal status		Partially met

*Continuous Professional Development

**The Qatar Counsel for Healthcare Practitioners

***continuous medical education

Study participants indicated that DG[w]5, which focuses on competency development, was not met because its three indicators were not achieved. The findings for DG[w] 5 suggest a lack of national finalized competency framework across all practice sectors in Qatar, which has reflected negatively on the first indicator of DG[w] 4.

DG[w] 6 has a focus on leadership development, and according to study participants this goal was partially met as two indicators were not achieved. One response about the only achieved indicator, by a professional leader from the MoPH, was:

“Qatar Council for Healthcare Practitioner has conducted few activities for the continuous profession development (CPD) teams of the CPD Providers to support in the educational planning of accredited CPD activities such as: Leadership Skills for CPD Professionals and Introduction to CPD Leadership course”. MoPH4

The study participants suggested that DG[w]7 (service provision and workforce education and training) was partially met with most of its indicators achieved through the systematic development of education and training activities based on local healthcare systems, their capacity and funding. For example, one of the MoPH’s policymakers said:

“The CPD accreditation system is intended to promote consistent quality and excellence in continuous medical education and professional development taking into account the national health profile, priorities, and workforce distribution”. MoPH3

The last goal in the professional development cluster (DG[w] 8) focuses on demonstrating the significance of pharmacists’ interprofessional education and collaboration with other healthcare professionals. The study participants indicated that this goal was partially met due to lack of evidence of policy formation that partnership development and engagement among healthcare professionals to achieve better health outcomes. One response from a professional leader in the MoPH about this goal stated:

“Although the National CPD program and accreditation system promotes interprofessional education of all health care practitioners including pharmacists, there is no clear policy formation evidence to guide the process”. MoPH2

### Systems cluster

**Fig. 3:**
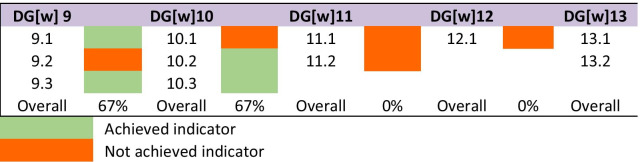
Percentages of indicators achievement in the systems cluster goals

All participants from MoPH responded to the systems cluster items. The systems cluster includes five goals, from which two goals were “partially met”, while the remaining three goals were “not met”. The three goals in the system cluster were not met due to unachieved indicators, lack of critical data, or because the goals were still under development stage. The detailed responses on the professional development cluster are shown in Table 4 and the percentages of the achieved indicators for each goal are shown in Fig. 3.

**Table 4:** Summary of the responses of the indicators in each goal among the systems cluster

DG[w] 9. Continuing professional development strategies: All professional development activity clearly linked with needs-based health policy initiatives and pharmaceutical career development pathways		
Indicator	Participants’ consensus about the indicator achievement status	Assessment of indicators
Evidence of an effective continuing professional development strategy according to national and local needs	CPD* strategies are designed to meet local needs	Achieved
Development of programs to support professional development across all settings of practice and all stages of a pharmacist’s career	Programs to support professional development are not well developed across all career stages yet	Not achieved
Education in continuing professional development strategies and self-directed behaviors should be initiated at the student level	Education is provided in these CPD* sessions, and self-directed learning is encouraged at student level. For working pharmacists, self-directed education is rewarded with CPD points for license renewal	Achieved

Overall goal status		Partially met
DG[w] 10. Pharmaceutical workforce gender and diversity balances: Clear strategies for addressing gender and diversity inequalities in pharmaceutical workforce development, continued education and training, and career progression opportunities		
Indicator	Participants’ consensus about the indicator achievement status	Assessment of indicators
Demonstration of strategies to address the gender and diversity inequalities across all pharmaceutical workforce and career development opportunities	A global benchmark for gender diversity for the pharmacy scope is needed to assess this goal	Not achieved
Ensure full and effective participation and equal opportunities for leadership at all levels of decision-making in pharmaceutical environments; avoidable barriers to participation for all social categories are identified and addressed	Effective participation at all levels for all social categories is ensured	Achieved
Engagement and adoption of workforce development policies and enforceable legislation for the promotion of gender and diversity equality; policies and cultures for the empowerment of all without bias	No gender bias exist across all policies of workforce engagement and development	Achieved

Overall goal status		Partially met
DG[w] 11. Workforce impact and effect on health improvement: Evidence of the impact of the pharmaceutical workforce within health systems and health improvement		
Indicator	Participants’ consensus about the indicator achievement status	Assessment of indicators
Engagement with systems to measure the impact of the pharmaceutical workforce on health improvement and healthcare outcomes Links with need-based education, training and workforce planning	Studies available in Hamad Medical Corporation (HMC), and this type of data is not in the Ministry of Public Health	Still needs further data. This indicator is considered as “Not achieved”
Gather continuous data points to monitor the performance of the pharmaceutical workforce	Not answered	Not achieved

Overall goal status		Not met
DG[w] 12. workforce intelligence: A national strategy and corresponding actions to collate and share workforce data and workforce planning activities (skill mixes, advanced and specialist practice, capacity). Without workforce intelligence data there can be no strategic workforce development		
Indicator	Participants’ consensus about the indicator achievement status	Assessment of indicators
Develop monitoring systems to identify workforce trends to enable decision-making on deployment and supply of pharmaceutical workforce noting that time-lags are present in these activities	The experts’ panel from the MoPH reported having a planned project to achieve these goals, but it is still in progress	Not achieved This indicator is currently Under development

Overall goal status		Not met
DG[w] 13. Workforce policy formation: Clear and manageable strategies to implement comprehensive needs-based development of the pharmaceutical workforce from initial education and training through to advanced practice		
Indicator	Participants’ consensus about the indicator achievement status	Assessment of indicators
Adopt and strengthen sound policies and enforceable legislation for holistic needs-based approaches to professional development across all settings and stages	The experts’ panel from the MoPH reported having a planned project to achieve these goals, but it is still in progress	Not achieved This indicator is currently Under development
Develop strategies where pharmaceutical science and professional services are the driving forces for this activity	These strategies are still in progress	Not achieved This indicator is currently Under development
Overall goal status		Not met

*Continuous Professional Development

Study participants suggested that DG[w] 9, which focuses on continuing professional development strategies, was partially met, because two indicators, which focuses on the effective continuing professional development strategies, were achieved. One participant’s response about these achieved indicators was:

“Qatar Council for Healthcare Practitioner continuous professional development activities’ accreditation standards mandate that all accredited activities must be planned to address the identified learning needs of the target audience. The needs assessment strategies include multiple sources of data to identify the needs of its target audience(s)”. MoPH3

Study participants indicated that DG[w]10, which addresses gender and diversity inequalities among the pharmacy workforce was partially met, because one of its three indicators were not achieved.

DG[w]11, which focuses on the workforce impact on health improvement, was not met, because one of its indicators needs further data to be exported from HMC, and the other indicator was not answered. However, one participant indicated the significance of this goal by stating:

“Participating in CPD activities would enhance the competence and performance of healthcare practitioners including pharmacists and, subsequently, ensure better healthcare service quality and patient care outcomes”. MoPH4

DG[w]12 has a focus on workforce intelligence and indicates that countries should have national strategies and corresponding actions to share workforce data and planning activities. Although this goal was not met directly, a profession leader in the MoPH commented that this goal is under development:

“The national continuous medical education/continuous profession development accreditation system promote stakeholder collaboration in CPD activity planning. In addition, the national needs-assessment strategy is planned to be formulated based on the available workforce data, perceived and unperceived educational needs assessment.” MoPH2.

**Fig. 4:**
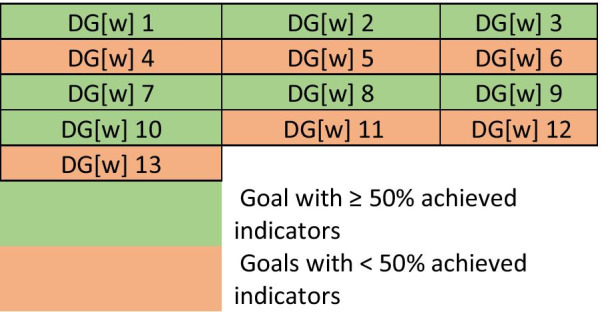
Degree of goals’ achievement among the three clusters

The study findings suggest that DG[w]13 (workforce policy formation) was not met, as the two principal indicators remain under national development. The degree of achievement for all goals among the three clusters is illustrated in Fig. 4.

## Discussion

This study is a preliminary step towards pharmacy education and workforce transformation in Qatar through evaluating the current situation of pharmacy education and workforce using the FIP workforce development goals as a mapping framework. The discussion of the results was conducted in relation to the limited number of similar studies conducted in response to the establishment of FIP’s roadmap and DG[w]s, as well as in relation to studies discussing elements similar to those covered in DG[w]s.

The study findings suggest that DG[w]s are interdependent and correlated; a certain gap in one goal generally was associated with gaps across other goals. Examples of correlation between various DG[w]s is demonstrated through the discussion of DG[w]1, DG[w]5 and DG[w]12 and their influence on DG[w]3, DG[w]6 and DG[w]1, respectively. This finding about the correlation between DG[w]s is consistent with the key results of the seven international workshops, which discussed the global transformative roadmap for pharmaceutical workforce and covered all six WHO regions [34].

The research findings regarding DG[w]1 suggest that there is an increase in academic capacity associated with establishing the CPH in 2007, which increase is not based on published national needs. This ‘capacity gap’ between reality and policy need could be rooted in communication gaps that potentially exist between the policymakers in the MoPH and the CPH in workforce planning. This in turn may have potential links with the quality assurance goal in the academy cluster (DG[w] 3), as key stakeholders in the MoPH potentially did not have adequate input on CPH’s policy development. The capacity measures for pharmacy students and graduates, which are not mapped to any published national needs, can be attributed to gaps in workforce intelligence and workforce planning (DG[w] 12 in the system cluster). Previous literature by Bader et al. indicated the importance of considering national needs when planning academic capacity (DG[w] 1) through a workforce intelligence system [35]. Bader et al. provided an overview of the pharmacy workforce capacity trends in the EMR and demonstrated that the EMR countries have relatively high workforce capacity compared to other WHO regions. However, EMR demonstrated several challenges in workforce intelligence planning compared to other regions, such as poor planning and management of human resources, geographic and sectoral distribution imbalances, limited educational and training capacities, workforce shortages, and underemployment and these findings are consistent with this Qatar study [35].

Results report that DG[w] 2 is partially met, which potentially suggests a general lack of strategies for the foundation training and early career development. This potential lack of strategies could be attributed to absence of the frameworks to support the transition of students into early career and towards advanced practice in the CPH. Several studies in the literature have emphasized the significant impact of early career development of novice pharmacists in building future career success and professional outcomes [36, 37]. For example, Koen et al. argued that early career training has improved the career adaptability, career success and career confidence among the newcomers to the labor market [36].

With regard to DG[w] 3, the participants suggested an implementation of internal and external quality assurance measures at CPH. In addition, participants indicated that DG[w]3 has been influenced by the capacity gap’ between national need and policy need (DG[w]1). In this regard, Bruno et al. highlights variability in pharmacy education and quality assurance measures at both the regional and global levels [34]. Bruno et al. argued that this variability is caused by lack of competency frameworks and quality assurance systems [34] which emphasizes the significance of developing such quality assurance measures for pharmacy education in the EMR generally [8].

In this study, the advanced and specialist expert development goal (DG[w] 4) was considered to be partially met, principally due to lack of common and shared understanding of specialization and advanced practice. This finding is consistent with one of the suggestions reported by pharmacy stakeholders in the international workshops that took place in November 2016, which discussed the global transformative roadmap for pharmaceutical workforce [34]. The suggestion emphasized the significance of financial resources for driving advanced practice and specialization, because remuneration and financial motives enhance the provision of advanced services [34].

Although DG[w]5 was not met due to a lack of any unified competency framework across all pharmacy practice sectors, national efforts are taking place to develop one. The significance of having a clear competency development framework was stressed in the literature as a priority for workforce development [38, 39]. Battel-Kirk et al. and Wright et al. argued that utilizing a competency-based approach is beneficial in identifying the required competencies, which can ultimately enhance the quality of practice as well as address complex and changing demands for critical services [38, 39]. It is worth noting that the absence of competency development frameworks (DG[w] 5), has influenced progress towards meeting indicators for DG[w] 6 (leadership development), because leaders, as well, needs to adhere to common competencies in their leadership practices, which are lacking. This also links to the progress of DG[w] 4. In that regard, Zilz et al. indicated the significance of developing the professional leadership skills and competencies in creating high-performance pharmacy practices characterized by a productive and quality patient care services [40].

For DG[w] 7, which was partially met through the systematic development of education and training activities based on local healthcare systems, several studies in the literature highlighted the value of implementing a systematic development among pharmacy workforce’s education and training approaches, based on social determinants [41, 42]. For example, Wheeler et al. (2014) argued that flexible pharmacist-related education and training programs are required by all community pharmacy staff, because these programs will improve pharmacists’ knowledge and build their confidence in service provision for patients [41]. However, it is worth noting that several global key pharmacy stakeholder respondents who attended the Global Conference on Pharmacy and Pharmaceutical Education reported varying interpretations of DG[w] 7 [34]. For example, ‘foundation training’ was considered by some stakeholders as initial education rather than workforce education and training, and by others to relate to early career training [34].

With regard to DG[w] 8, the literature emphasized the significance of partnership development among healthcare professionals to achieve better health outcomes, which was partially met in this study. For example, Farrell et al. and El-Awaisi et al. stressed the value of interprofessional education and collaborative working on providing patient-centered care and in initiating a system-level intervention in order to improve healthcare outcomes [43, 44].

DG[w] 9 was considered to be partially met, but was classed by participants as a priority for development, indicating that all accredited activities must be planned to address the identified learning needs of the target audience. Several published studies have emphasized the significance of continuing professional development for the pharmacy workforce and considered it as an ethical requirement in different healthcare professions [45, 46]. Furthermore, continuing professional development for the pharmacy workforce is a commitment towards patients and quality healthcare delivery, and pharmacists should regularly engage with continuing professional development to apply advanced guidelines and strategies in their career [45, 47].

DG[w]10, that focuses on pharmaceutical workforce equity and diversity balances, was partially met with two of its indicators achieved. In that regard, the literature highlighted the importance of clear strategies to address gender and diversity balance across pharmacy workforce and to consider them in career development efforts [48–50]. Moreover, several studies indicated the significance of removing any barrier that negatively impact the active participation of all pharmacy workforce [48–50], which would ultimately improve the overall status of DG[w]10. For DG[w]11 (workforce impact and effect on health improvement), which was not met in this study because one of its indicators needs further data to be exported from HMC, several studies have illustrated the impact of the pharmaceutical workforce on health improvement [51, 52]. For example, Basheti et al. indicated that the pharmaceutical workforce has a key role in improving patients’ health outcomes in Jordan [51]. Furthermore, the value of pharmacy workforce interventions were considered as highly valuable in several countries [53, 54].

According to the findings, DG[w] 12, which focuses on workforce intelligence, was considered under development. A key obstacle in achieve DG[w] 12 in the State of Qatar is the absence of a national pharmacy association that represents and supports pharmacists nationally and internationally. The mentioned gap in workforce intelligence and workforce planning has influenced DG[w] 1, which focuses on capacity measures for pharmacy students and graduates.

This obstacle has been indicated in several previous studies that discussed the pharmacy profession and practice in Qatar [11, 55]. For example, Kheir and Fahey stressed the value of having a pharmacy association or agency in Qatar to enhance the transformation of pharmacy professional practice and to provide guidance [11]. In addition, the significance of having such associations or agencies for planning and developing workforce was stresses in several previous literature from different regions [56, 57]. Although pharmacy law in Qatar has a key role in controlling pharmacies, regulating pharmacists’ registration process, checking the structure of the pharmacies’ premises, and regulating medications’ use regulation, yet the key role of a national pharmacy association is non-replaceable.

Finally, DG[w] 13 was considered “not met” because the unified policies and strategies for professional development across all settings and stages are still under development. In that regard, a global report published in 2017 by the FIP emphasized the importance of having implementation plans to meet national-level pharmaceutical workforce strategies [58]. The report indicated that 21 countries reported on their progress towards achieving the 13 DG[w]s, and emphasized that policy and strategy formation and workforce intelligence (DG[w] 12 and 13) were key challenges for almost all countries [58].

The findings reported in this study offer an important first step in identifying and prioritizing gaps in pharmaceutical workforce planning and development in Qatar, and in encouraging aligned future research in other EMR countries. Such national–regional engagement activities would result in an opportunity to enhance EMR-wide efforts to strategically progress development of higher quality education and practice standards, and would offer an evidence-led case study for other WHO regions [23]. Moreover, overcoming the identified gaps in this study would ultimately contribute to the successful achievement of the third pillar of Qatar’s 2030 vision, which aims to build a comprehensive world-class healthcare system that is effective, affordable and universally available to all citizens [20].

One of the study’s limitations is lacking data from the pharmacy practice sectors whether governmental or private. However, since the MoPH is the governing body responsible for both private and public practice sectors, the provided data from MoPH participants was considered representative of the current situation among practice sectors in Qatar. Further research should focus on collecting data from the governmental and private practice sectors in order to represent a full image for the pharmacy practice in Qatar. Indeed, establishing a national pharmacy association in Qatar would ultimately result in the appropriate representation, monitoring, and reporting all pharmacy practice-related sectors’ status and concerns.

Another limitation for this study is the difficulty in how some DG[w]s and their associated indicators are understood and interpreted. This limitation can be attributed to a lack of universally accepted terminology or glossary of terms related to DG[w]s. Furthermore, DG[w]s and the utilized survey tool are newly developed and published (2017) and there is a dearth in the literature that can be used to compare and interpret the results of this study. This observation is supported by the summary note published by the key pharmacy stakeholders in the FIP, who attended the Global Conference on Pharmacy and Pharmaceutical Education. They suggested having a “terminology and definitions framework” to tackle different contextual meanings of the goals [34]. To address this limitation, researchers in this study met with FIP leaders to make sure that they fully understand the goals and the survey. Later on, the researchers met personally with the study participants and gave them a summary of each goal to ensure the proper understanding of the goals before giving their consensus on responses.

Furthermore, the research involved a small number of participants from MoPH and CPH (6 participants and 5 participants, respectively). However, those participants were the key decision-makers in pharmacy practice from MoPH and policymakers and leading educators in the CPH at QU.

## Conclusions

This research was the first conducted in the State of Qatar and in the Eastern Mediterranean Region to evaluate the current situation of pharmacy education and practice and its alignment with the global Development Goals (workforce) in the State of Qatar. The DG[w]s developed by the FIP and global consensus have provided a transnational opportunity and integrated platform for comprehensive workforce development. This study has demonstrated its effectiveness in identifying and examining the current gaps of the pharmacy workforce in Qatar. This study has assessed the country-level context of the global DG[w]s in three clusters: academy, professional development, and systems. The results suggest that the disconnect between academic capacity planning and national health resources needs, the lack of evidence-based competency development in all professional career stages, and the undeveloped workforce policy and workforce intelligence are significant workforce development vulnerabilities and should be identified as priority policy gaps.

Future multinational research should focus on optimizing the terminologies and definition of frameworks which are being used globally to address the DG[w]s, in order to reach a better common shared understanding by all pharmacy educators, professionals and leaders around the world. Furthermore, based on our findings, we conclude that EMR countries should find ways of collaborating, in a transnational way, in order to enhance efficiencies in policy development identified by our study; in this way, the gaps in pharmacy education, practices and policies can be uniformly prioritized to initiate concerted workforce planning and prevent shortages or oversupply of pharmacists in all professional sectors. Additionally, future research should be conducted in Qatar in order to understand the practice sector vision and perspective with regard to relevant DG[w]s.

## Data Availability

The datasets used and/or analyzed during the current study are available from the corresponding author on reasonable request.

## Appendix

See Table 5.

**Table 5:** The Pharmaceutical Workforce Development Goals description

Cluster	DG[w]	DG[w] general description
Academy Focus on the schools, universities and education providers	1. Academic capacity	Foundation training infrastructures in place for the early post-registration (post-licensing) years of the pharmaceutical workforce as a basis for consolidating initial education and training and progressing the novice workforce towards advanced practice
	2. Foundation training and early career development	
	3. Quality assurance	Transparent, contemporary and innovative processes for the quality assurance of needs-based education and training systems
Professional development Focus on the pharmaceutical workforce	4. Advanced and specialist expert development	Education and training infrastructures in place for the recognized advancement of the pharmaceutical workforce as a basis for enhancing patient care and health system deliverables
	5. Competency development	Clear and accessible developmental frameworks describing competencies and scope of practice for all stages of professional careers. This should include leadership development frameworks for the pharmaceutical workforce
	6. Leadership development	Strategies and programs in place that develop professional leadership skills (including clinical and executive leadership) for all stages of career development, including pharmaceutical sciences and initial education and training
	7. Service provision and workforce education and training	A patient-centered and integrated health services foundation for workforce development, relevant to social determinants of health and needs-based approaches to workforce development
	8. Working with others in the healthcare team	Clearly identifiable elements of collaborative working and interprofessional education and training which should be a feature of all workforce development programs and policies
Systems Focus on policy development, governmental strategy and planning, and monitoring systems	9. Continuing professional development strategies	All professional development activity clearly linked with needs-based health policy initiatives and pharmaceutical career development pathways
	10. Pharmaceutical workforce gender and diversity balances	Clear strategies for addressing gender and diversity inequalities in pharmaceutical workforce development, continued education and training, and career progression opportunities
	11. Workforce impact and effect on health improvement	Evidence of the impact of the pharmaceutical workforce within health systems and health improvement
	12. Workforce intelligence	A national strategy and corresponding actions to collate and share workforce data and workforce planning activities (skill mixes, advanced and specialist practice, capacity). Without workforce intelligence data there can be no strategic workforce development
	13. Workforce policy formation	Clear and manageable strategies to Implement comprehensive needs-based Development of the pharmaceutical workforce from initial education and training through to advanced practice

## Abbreviations

FIP: International Pharmaceutical Federation
DG[w]s: The Pharmaceutical Workforce Development Goals
MoPH: Ministry of Public Health
CPH: College of Pharmacy
QU: Qatar University
WHO: World Health Organization
EMR: Eastern Mediterranean Region
CPD: Continuous profession development

## Footnotes

1 This manuscript will refer to the principal 13 Workforce Development Goals as “Development Goals (workforce)” or DG[w] throughout.
